# Supplementary material for: The sphingolipids ceramide and inositol phosphorylceramide protect the Leishmania major membrane from sterol-specific toxins
Source: J Biol Chem. 2023 Apr 23;299(6):104745. doi: 10.1016/j.jbc.2023.104745 (PMC10209034; doi:10.1016/j.jbc.2023.104745)

Supplementary Figure S1

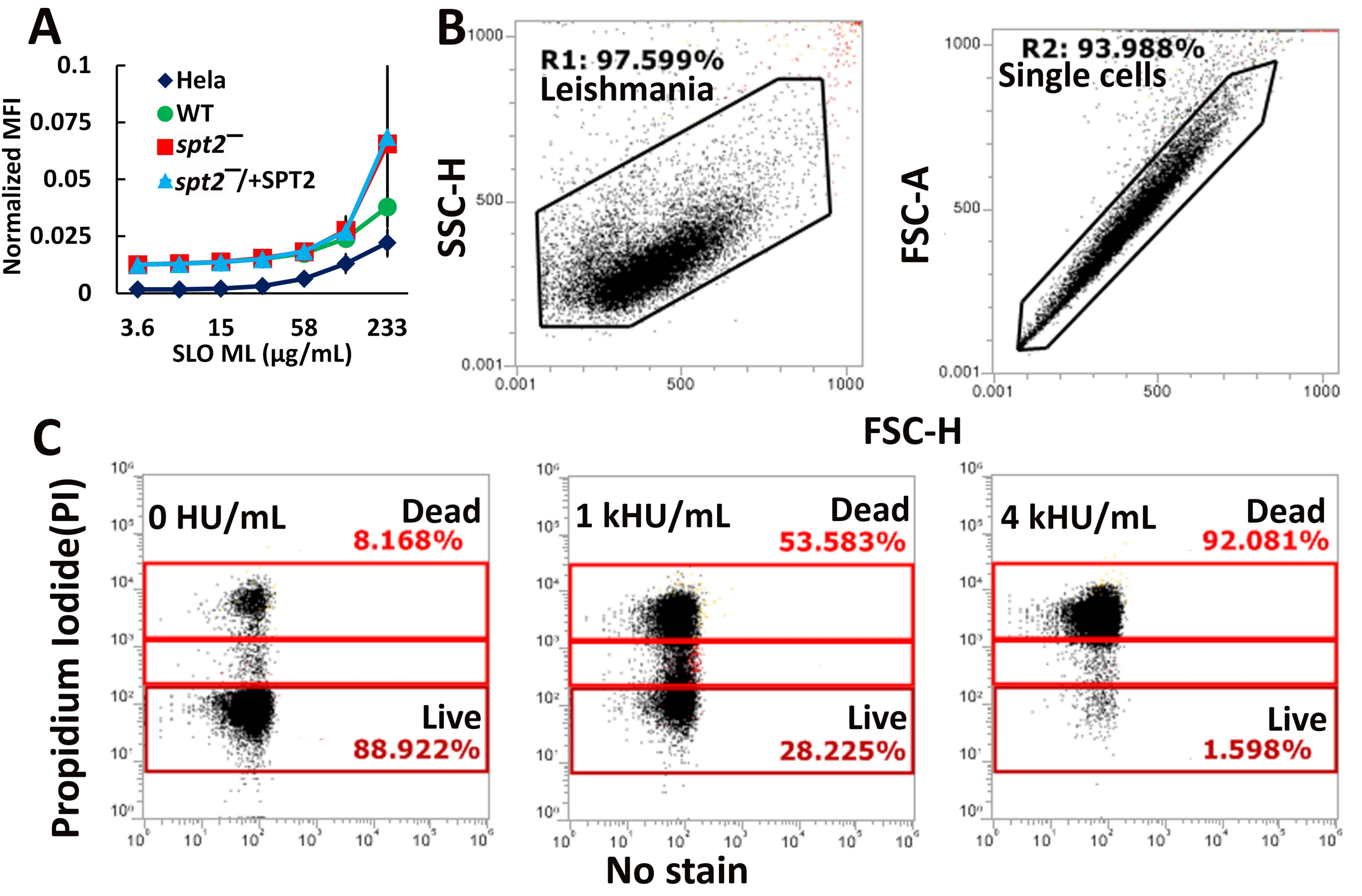

Supplementary Figure S2

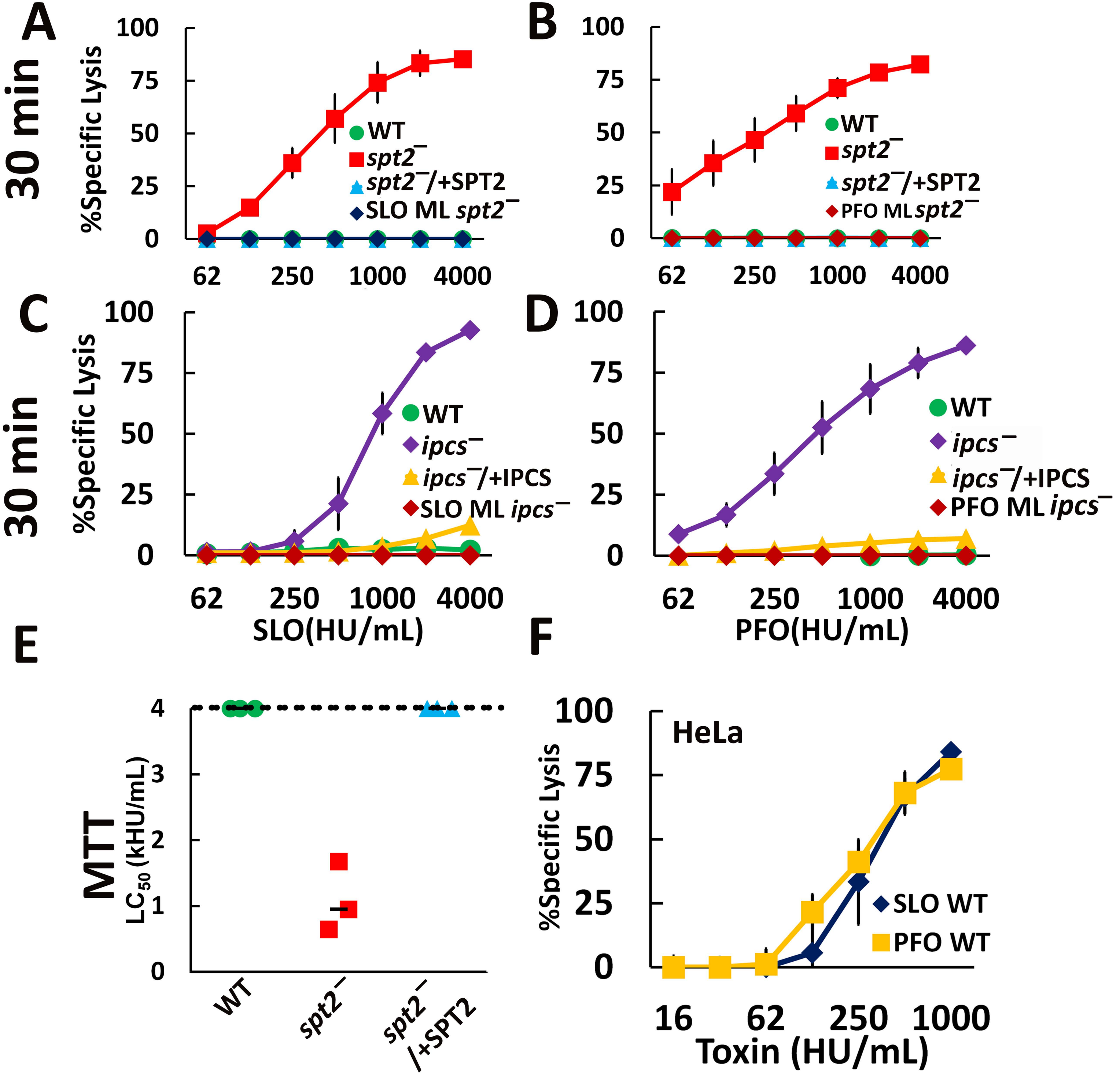

Supplementary Figure S3

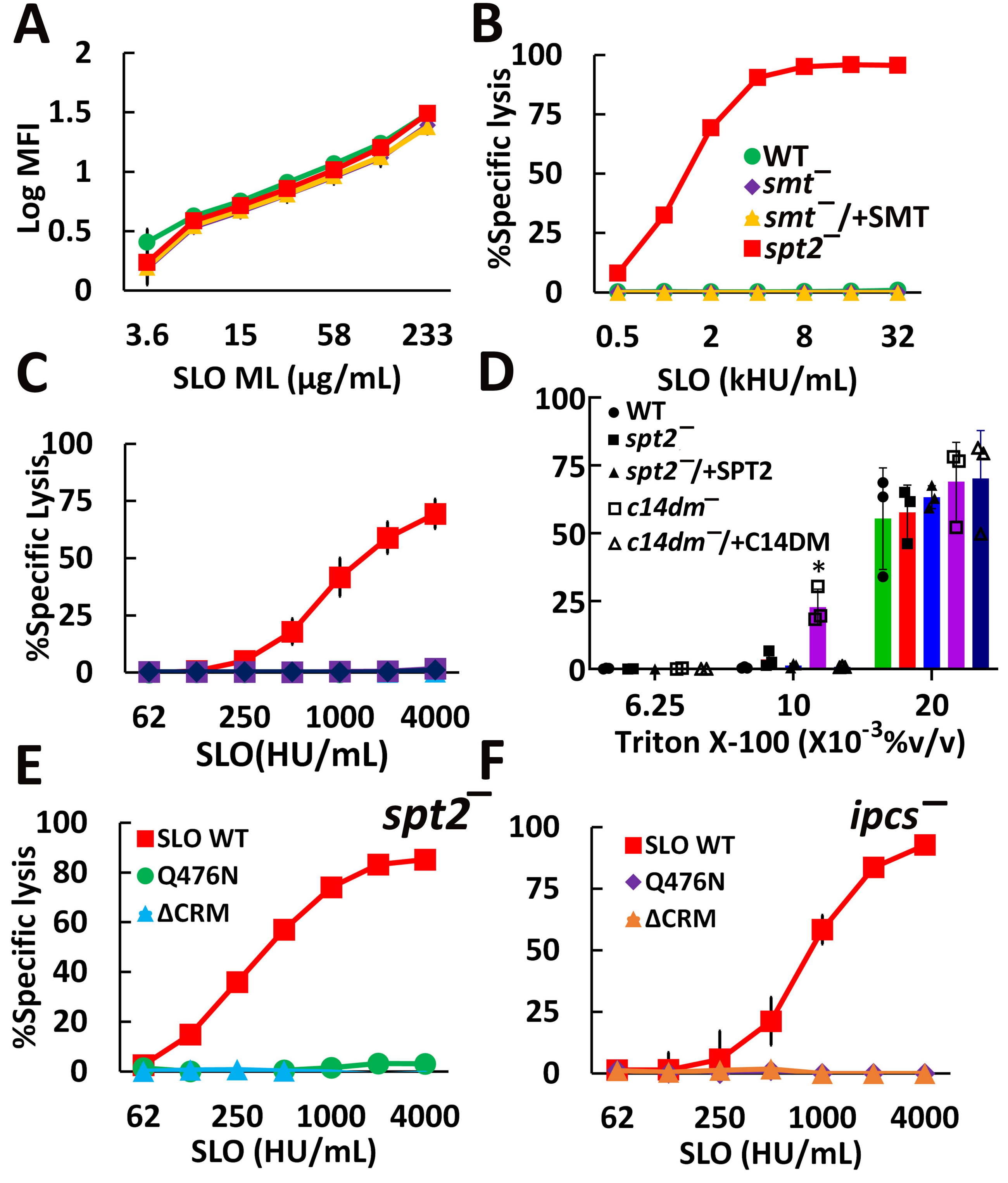

Supplementary Figure S4

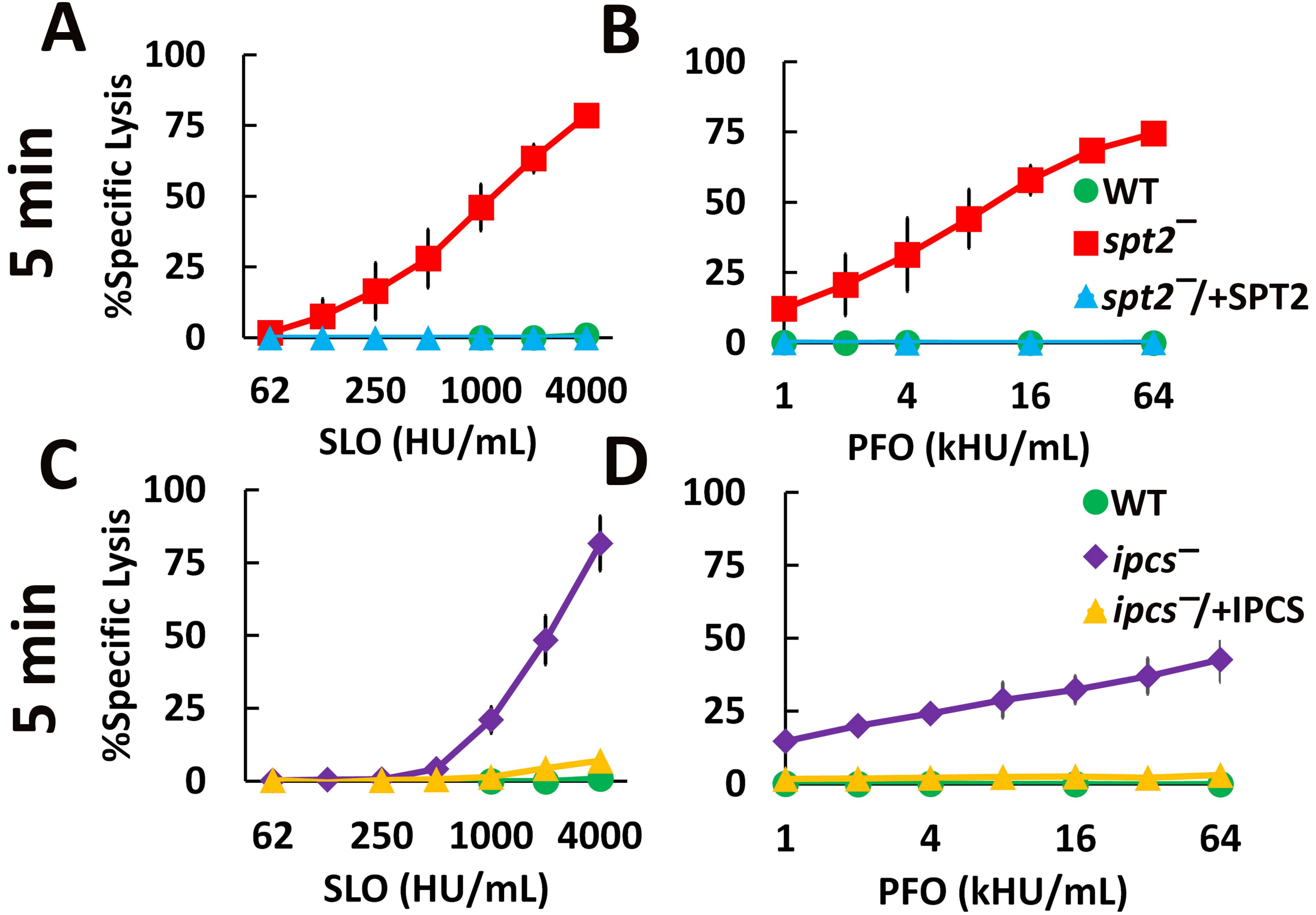

Supplementary Figure S5

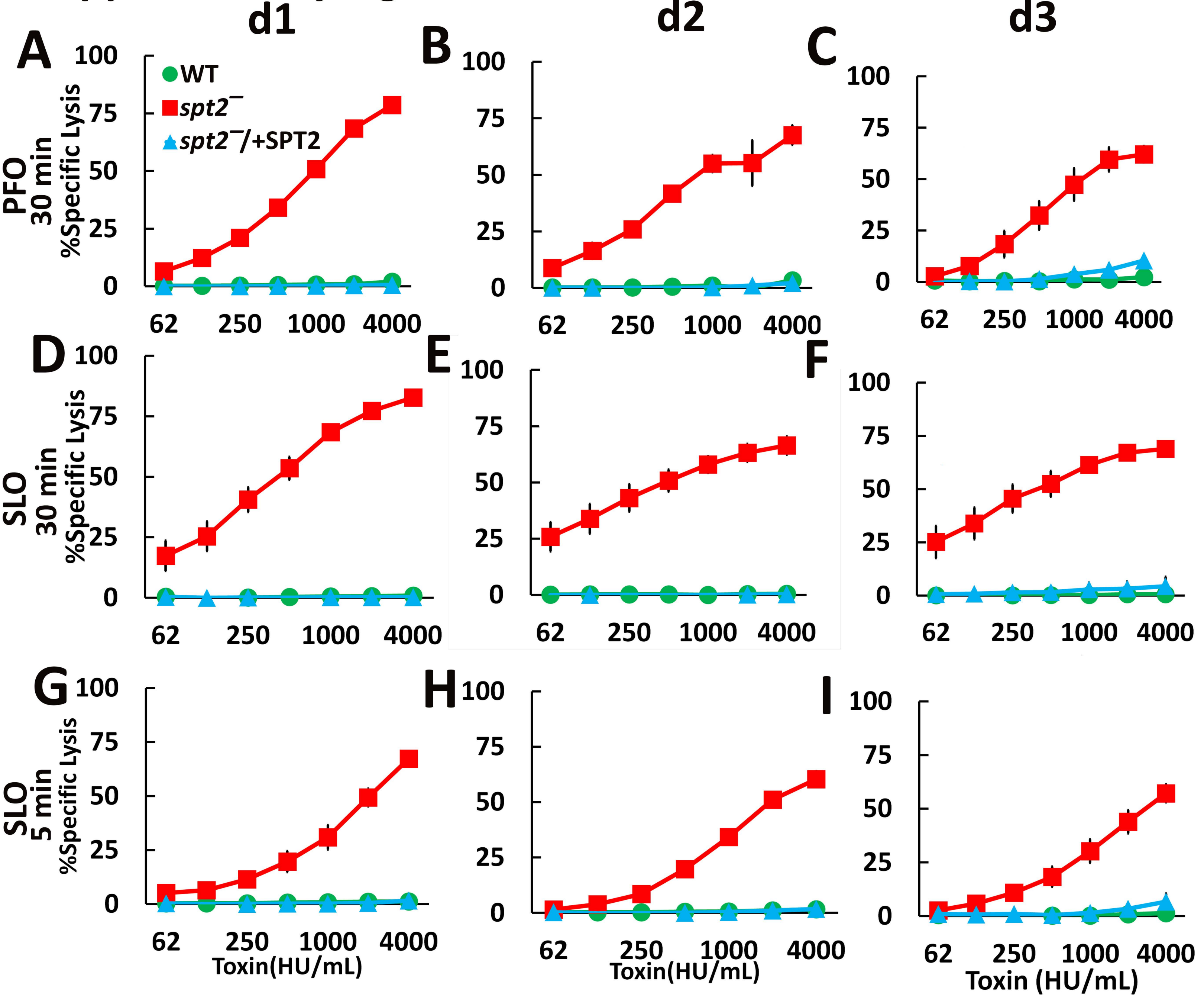

Supplementary Figure S6

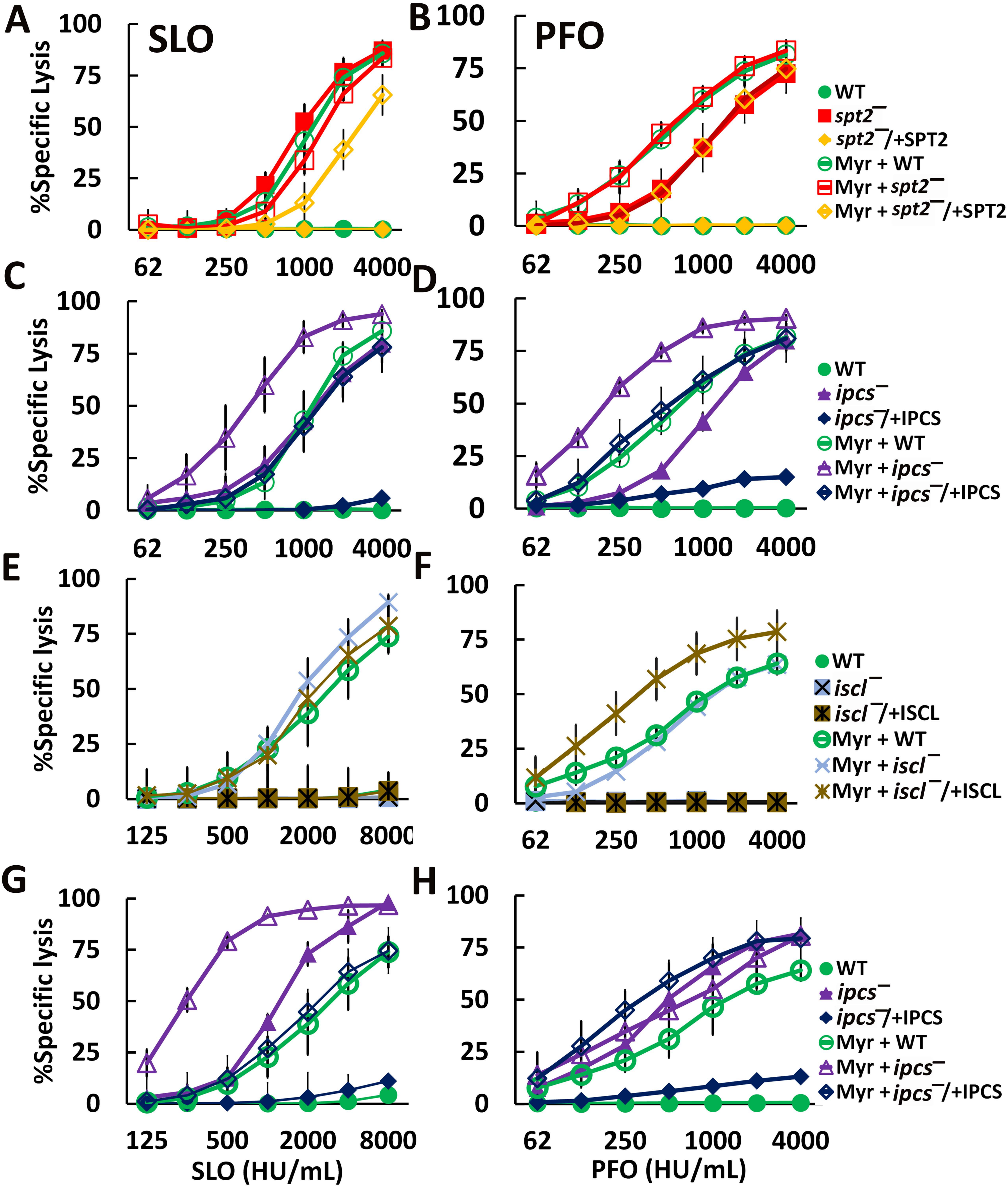

Supplementary Figure S7

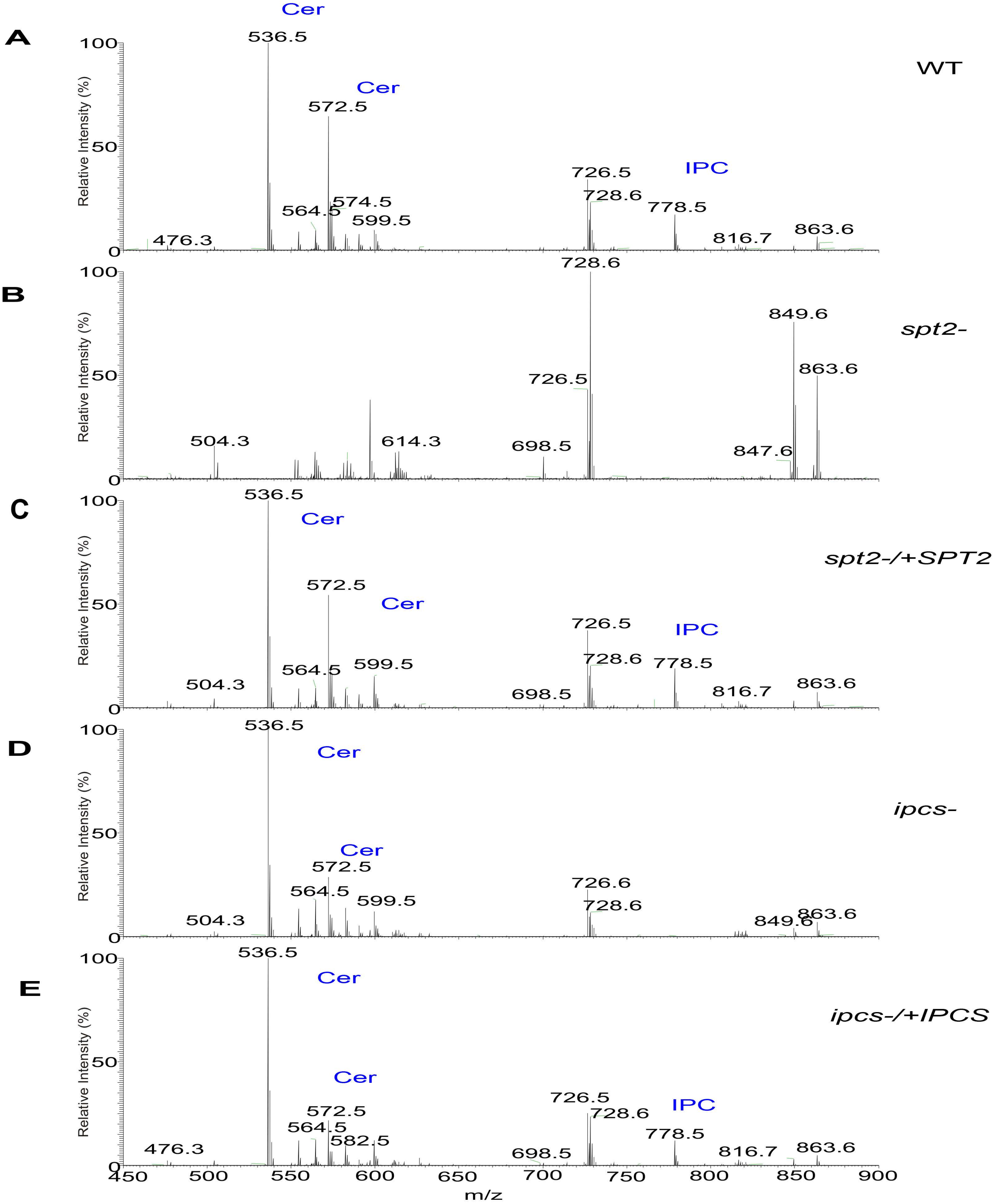

Supplement: Supporting Figure S1 — Binding analysis and gating strategy for Leishmania major promastigotes.A, The MFI from Fig 1F was normalized by cell surface area, assuming a sphere of radius 7.5 μm for HeLa cells in suspension and an ellipsoid calculated using the Knud-Thompson formula with a = 5 μm and b = c = 1.25 μm. MFI was divided by surface area. B, Total Leishmania major promastigotes are gated on R1 gate for SSC-H and FSC-H. L. major promastigotes are then gated for single cells (R2) using FSC-A and FSC-H. C, From R2, L. major promastigotes are gated for fluorescence intensity of propidium iodide (PI) for dead cells and live cells. A, The x-axis is a log2 scale. C, Both axes are log10 scale Supporting Figure S2: Pore-formation, glycan- and sterol- binding determinants are all required for cytotoxicity in Leishmania major promastigotes.A and B, Wild type (WT), spt2—, and spt2—/+SPT2, or (C and D) WT, ipcs—, and ipcs—/+IPCS L. major promastigotes were challenged with (A) monomer-locked SLO (SLO ML), (B) monomer-locked PFO (PFO ML), (A and C) SLO or (B and D) PFO at the indicated concentrations for 30 min at 37 °C and PI uptake measured by flow cytometry. E, WT, spt2—, and spt2—/+SPT2 L. major promastigotes were challenged with 62-4000 HU/mL SLO for 30 min at 37 °C and viability measured by MTT assay. Analysis is described in the methods. Points on the dotted line had an LC50 > 4000 HU/mL. F, Hela cells were challenged with SLO WT or PFO WT at the indicated concentrations for 30 min at 37 °C and PI uptake measured by flow cytometry. (A–D and F) The x-axis is a log2 scale Supporting Figure S3: Sterol alterations and general membrane perturbations do not account for cytotoxicity.A and B, WT, smt—, and smt—/+SMT, or spt2—L. major promastigotes were challenged with (A) SLO ML conjugated to Cy5 or (B) SLO at the indicated concentrations for 30 min at 37 °C and PI uptake measured by flow cytometry. E, spt2— and (F) ipcs—L. major promastigotes were challenged with SLO (WT), SLO Q476N or SLO ΔCRM at [file mmc1.pdf]
